# Mental health disorders among medical students during the COVID-19 pandemic in the area with no mandatory lockdown: a multicenter survey in Tanzania

**DOI:** 10.1038/s41598-024-53885-5

**Published:** 2024-02-11

**Authors:** Deogratius Bintabara, Joseph B. Singo, Mathew Mvula, Sichone Jofrey, Festo K. Shayo

**Affiliations:** 1 Department of Community Medicine, School of Medicine and Dentistry, The University of Dodoma, Dodoma, Tanzania; 2 Tanrep Research Consultancy Limited, Dar es Salaam, Tanzania; 3 Department of Internal Medicine, Muhimbili National Hospital, Dar es Salaam, Tanzania

**Keywords:** Depression, Anxiety, Stress, Medical students, COVID-19 pandemic, Tanzania, Viral infection, Anxiety, Depression

## Abstract

The COVID-19 pandemic brought about a major public health concern worldwide. It forced many countries to enforce lockdowns, leading to the closure of higher learning institutions. The abrupt shift in the lifestyle of students had a profound impact on their mental health. This study aims to determine the prevalence and factors associated with mental health conditions among university students in Tanzania during the COVID-19 pandemic. A sample of 425 students from six medical universities and colleges in Tanzania completed an online survey and was included in the analysis. The questionnaire consisted of validated Depression, Anxiety and Stress Scale—21 Items (DASS-21) questions (Cronbach’s alpha = 0.92) assessing the presence of mental health symptoms: depression, anxiety, and stress. Multivariable logistic regression models were fitted to explain the factors associated with mental health conditions. A *P*-value < 0.05 was considered statistically significant in all inferential analyses. The median age (interquartile range) of the participants was 24 (22–26). The prevalence of mental health conditions was 28.94%, 54.12%, and 15.06% for depression, anxiety, and stress, respectively, while the prevalence of having any mental health condition was 58.59%. In an adjusted regression model, being in the fourth and fifth years of study and living with a spouse were significantly associated with increased odds of depression: AOR = 5.99 (1.31–27.47), AOR = 5.52 (1.18–25.81), and AOR = 1.84 (1.08–3.15), respectively. Moreover, studying in private universities and living with a spouse were significantly associated with an increased likelihood of anxiety: AOR = 2.35 (1.72–2.76), and AOR = 2.32 (1.20–4.50), respectively. The likelihood of stress was only among participants studying in private universities; AOR = 2.90 (1.60–5.27). The study revealed alarmingly high rates of mental health conditions among medical students in Tanzania during the COVID-19 pandemic. The findings suggest the need for regular checkups for medical students regarding their mental health status. Additionally, it recommends that the government and other stakeholders establish mental health services within the universities for the effective prevention of the rising burden of mental health problems among universities in Tanzania and other countries with similar settings.

## Introduction

The spread of the coronavirus disease 2019 (COVID-19) pandemic worldwide in early 2020 had an immense impact on all socioeconomic sectors, including education^1^. Following the declaration of the COVID-19 pandemic in March 2020 by the World Health Organization (WHO), world leaders were faced with the challenge of maintaining the normal operation of different sectors amid the pandemic^2^. The first approach used by both heavily and minimally affected countries was to impose a total or partial lockdown in order to minimize the spread of COVID-19^3^. Education institutions, including universities and colleges, were among the most affected as the students were caught by surprise with the sudden halt of classes and the requirement to stay at home for an unknown period of time^1,4,5^. However, in most developed countries, teaching and assessments were shifted online^1^. This was not the case in most developing countries due to the lack of developed and resilient digital technology at universities^4,5^. As a results, students in developing countries faced uncertainty regarding the continuation of their studies and when they would be able to resume their academic life^6^.

During the COVID-19 pandemic, the younger population including university students were clinically not regarded as a risky or vulnerable population, however, there was a great concern about their mental health as a result of the pandemic^7–10^. To date, in various parts of the world, especially in developed countries, studies have shown that there is an increase in mental health conditions, such as stress, anxiety, and depression^11–17^. Given the fact that mental health conditions among university students were also prevalent even before the COVID-19 pandemic^18–20^, the evidence shows that the pandemic has increased its prevalence and magnitude^21,22^.

Since the onset of the COVID-19 pandemic, the study of mental health among students has been given great attention in social science research. Various studies conducted around the globe have documented the detrimental effect of the pandemic on diverse aspects of mental well-being among students of from diverse groups. According to these studies, stress, anxiety, and depression were the main mental health conditions reported among students in higher learning institutions during the pandemic^23^. For instance, a cross-sectional study conducted in countries of Eastern Europe, the Middle East, and South America, involving 2349 university students revealed that the average prevalence of stress, depression, and generalized anxiety symptoms was 61.3%, 40.3%, and 30%, respectively^12^. In the United Kingdom, one study found that more than 50% of university students experienced high levels of anxiety and depression above the clinical cut-offs. This was due to low levels of resilience as a result of lockdown-related isolation and restrictions^13^. In China, a large cross-sectional survey during early 2020 among 821,218 college students reported that the prevalence rates of acute stress, depression, and anxiety symptoms were 34.0%, 21.1%, and 11.0, respectively^14^. Furthermore, a systematic review and meta-analysis of cross-sectional studies in China revealed that the pooled prevalence of anxiety symptoms was 25.0% (95% CI 21–29%, p < 0.001) among college students^15^. In Africa, there have been few studies that reported the mental health impact of to COVID-19 pandemic among university students^24,25^. In South Africa, the prevalence of anxiety and depression during lockdown was 45.6% and 35.0%, respectively^16^. In Uganda, one study reported a significantly higher prevalence of depression (80.7%), anxiety (98.4%), and stress (77.9%) as a result of the pandemic^17^.

The majority of the research on the mental health impact due to COVID-19 pandemic has been carried out in Europe, China, and North America compared to African countries including Tanzania^24,25^. Furthermore, few African countries including Tanzania imposed partial or total lockdowns in the first wave of the pandemic and lifted the lockdown in the subsequent waves^3^. This was because the federal government was unable to support and maintain the socioeconomic activities of the citizens due to poverty^3^. In this accord, together with other factors, the prevalence and magnitude of mental health among students of higher learning institutions due to the impact of the pandemic in African countries have been different from other countries across the world^25,26^.

Tanzania had a unique approach to the COVID-19 pandemic: a brief total lockdown in the first wave of the pandemic followed by unscientific denial of the pandemic and resuming of normal life^27–29^. The denial of the existence of the pandemic had a strong political influence and enforcement and hence it was very difficult to conduct social science studies on the impact of the COVID-19 pandemic^27–29^. It was then, in late 2021 when the government re-declared the existence of the pandemic and implemented the rollout of the COVID-19 vaccines^30^. Therefore, the impact of the COVID-19 pandemic on mental health among university students in Tanzania can have a different picture if compared to other countries context within Africa and across the world. To date, less is known about the impact of the COVID-19 pandemic on the mental health of the general population including students in higher learning institutions in Tanzania. Therefore, the current study sought to determine the prevalence and the factors associated with mental health conditions among university students in Tanzania during the COVID-19 pandemic.

## Materials and methods

### Study design

A descriptive-analytical cross-sectional study (online survey) was conducted from January to March 2022.

### Study participants and study setting

Medical students were recruited from six (three public and three private) medical universities in Tanzania. The public universities are (i) The University of Dodoma (UDOM), (ii) Muhimbili University of Health and Allied Sciences (MUHAS), (iii) University of Dar Es Salaam (UDSM-Mbeya) while the private universities are (iv) Catholic University of Health and Allied Sciences (CUHAS), (v) Hubert Kairuki Memorial University (HKMU) and (vi) St. Francis University College of Health and Allied Sciences (SFUCHAS). These medical universities are located in the five major cities in Tanzania which are Dar es Salaam, Dodoma, Mbeya, Morogoro, and Mwanza.

### Sample size and sampling procedures

The sample size was determined by using a single population proportion by taking the prevalence of mental distress 45.9%; a study reported from University of Gondar, Northern Ethiopia^31^ with; a 5% margin of error, 95% confidence, population size of 5010 and assuming 20% nonresponse rate. Finally, a sample size of 426 medical students were achieved.

### Recruitment, enrolment, and data collection

In Tanzania, each medical university enroll about 135 to 200 student per year (an average of 167 students per year). As medical school in Tanzania taking 5 years, therefore a total average of 835 students per each university was estimated. For the six universities involved in this study brings a total population size of 5010 medical students. To determine the representative sample, authors considered sending email invitation to at least 1/10th of the medical students in Tanzania (0.1 × 5010 = 501). Therefore. email invitations with a link to a voluntary, de-identified survey were sent to 501 medical students. The information from all medical students remained anonymous to ensure the confidentiality and reliability of the data. Medical students who accepted the invitation to take part in the study clicked the link to complete the online questionnaire.

### Data collection and tools

The online questionnaire consisted of three sections; university students’ demographic information, academic profile (type of university and students’ current academic year), and information regarding symptoms of mental health disorders during the COVID-19 pandemic., DASS 21 questions were used to assess for the presence of symptoms related to mental health disorders. The original DASS comprised 42 items on a four-point Likert scale to assess the negative emotional states of depression, anxiety, and stress, with 14 questions for each subscale. However, Henry and Crawford in 2005, developed and validated a shorter version of the DASS, known as DASS 21, which comprised 21 items, where each of its sub-scales of depression, anxiety, and stress contained seven items.

The online English version of self-administered questionnaires was used during data collection because the study population used the English language which is the media of communication for medical training in Tanzania. The use of online questionnaires helps simplify the process of data collection as it saves time, is cheap, more affordable, and easily accessible.

This questionnaire included two sections. The first part contained the personal characteristics of the study participants such as age, marital status, year of the study, name of university, etc. The second section included information on the short form of the Depression, Anxiety, and Stress scale (DASS-21) which consists of 21 items that have been divided into three subscales of anxiety, stress, and depression, each with seven items. The subscale for depression assessed inadequacy, dissatisfaction, hopelessness, devaluation, and inertia. The subscale for anxiety is used to assess acute responses to fear as well as physical and mental symptoms of anxiety, while the stress subscale evaluates tension, restlessness, irritability, and difficulty relaxing. Questions 3, 5, 10, 13, 16, 17, and 21 form the depression scale; questions 2, 4, 7, 9, 15, 19, and 20 form the anxiety scale while questions 1, 6, 8, 11, 12, 14, and 18 are covered in stress scale. The sum of the scores for each sub-seven scale’s items was used to assess the presence and absence of depression, anxiety, and stress. The presence of depression, anxiety, and stress were indicated by a sum of scores ≥ 10, ≥ 8, and ≥ 15 respectively. The details regarding DASS 21 cut-off points for conventional severity labels are indicated in Table 1.

**Table 1:** Summary of suggested cut-off scores for conventional severity labels in DASS 21.

Severity	Depression	Anxiety	Stress
Normal	0–9	0–7	0–14
Mild	10–13	8–9	15–18
Moderate	14–20	10–14	19–25
Severe	21–27	15–19	26–33
Extremely severe	28+	20+	34+

### Validity and reliability of DASS 21

The reliability was tested by calculating the overall Cronbach’s alpha of the DASS 21 which was 0.92, indicating excellent internal consistency, while the Cronbach’s alpha of each sub-scale (Depression = 0.76, anxiety = 0.86, and stress = 0.87 (Supplementary Table 1).

### Measurement of variables

#### Outcome variables

Mental health disorders were considered if study participants were identified to have symptoms related to (i) Depression, (ii) Anxiety, and (iii) Stress based on DAS 21. Each of these three had a sub-seven scale containing seven factors rated from 0 to 3 on a Likert scale (0: “Did not apply to me at all,” 1: “Applied to me to some degree or some of the time,” 2: “Applied to me to a considerable degree or a good part of the time,” and 3: “Applied to me very much or most of the time”).

Medical students who fall under moderate, severe, or extremely severe groups in each sub-seven scale were considered as having depression, anxiety, or stress. In addition, medical students with any of these three were regarded as having mental health disorders.

#### Independent variables

In this study age was grouped into four categories which are “18–21,” “22–25,” “26–29” and “30 or more”. Year level of study was grouped into “first,” “second,” “third,” “fourth,” and “fifth”. Marital status was categorized as “single” and “married/cohabiting”. The university type was categorized as “public” for government-owned universities and “private” for private and faith-based universities. Clinical rotation “Yes” for medical students in the fourth or fifth year (attached in the hospital) and “No" for first, second, and third year who doing basic sciences. Lastly, vaccination status was coded as vaccinated for those who reported having been vaccinated with any type of vaccine against COVID-19.

#### Data processing and statistical analysis

Data was cleaned, edited, coded, and analyzed by using STATA version 17. During descriptive analysis, continuous variables were summarized using median and interquartile (IQR) while categorical variables were summarized using proportions, then presented in tables and graphs. Furthermore, a series of individual unadjusted logistics regression analyses were constructed across all three outcome variables; depression (model 1a), anxiety (model 2a), and stress (model 3a). Thereafter, all independent variables that showed an association with P < 0.2 from each unadjusted model were eligible for inclusion in their corresponding multivariable logistics regression analyses (model 1b, 2b, and 3b). All models were fitted using a stepwise (backward) elimination method and P < 0.05 was taken to indicate statistical significance. The odds ratio (OR) and 95% confidence interval (95% CI) for each variable were computed and used to measure the association with the outcome variables.

### Ethical statement

The ethical approval to conduct this study was obtained from the Ethical Research Committee of the University of Dodoma (reference MA.84/261/02 dated 17th January 2022). All study participants signed written informed consent and agreed to the publishing of their anonymized data. This study did not expose study participants to unnecessary risks. Confidentiality was kept at all levels of the study; it was assured by excluding names and identifiers in the questionnaire and the data were used only for this study purpose. The study was conducted by the guidelines and regulations of the Declaration of Helsinki.

## Results

### Respondents’ characteristics

As presented in Table 2, out of 501 who were invited a total of 425 medical students responded, completed the online questionnaire and their information was included in the analysis. The median age (IQR) of the respondents was 24 (22–26) years. More than a quarter (27.3%) of the respondents were living with their spouse at the time of the pandemic. Nearly 80% of the respondents were from publicly owned universities. Furthermore, more than half of the respondents reported having been vaccinated against COVID-19.

**Table 2:** Percent distribution of medical students by selected background characteristics, Tanzania, 2021 (n = 425).

Variable	n (%)
Age (median (IQR) = 24) (23–26)	
18–21	29 (6.82)
22–25	271 (63.76)
26–29	100 (23.53
≥ 30	25 (5.88)
Year level of study	
First	23 (5.41)
Second	46 (10.82)
Third	90 (21.18)
Fourth	131 (30.82)
Fifth	135 (31.76)
Marital status	
Single	309 (72.71)
Married/cohabiting	116 (27.29)
University type	
Public	333 (78.35)
Private	92 (21.65)
Clinical rotation	
No	129 (30.35)
Yes	296 (69.65)
Vaccination status	
Unvaccinated	115 (43.89)
Vaccinated	147 (56.11)

### Prevalence of mental health disorder

Table 3 shows the percentage distribution of depression, anxiety, stress, and any form of mental health disorders among medical students in Tanzania. Most of the respondents presented with normal ranges for depression (44%), anxiety (36%), and stress (77%) compared to other ranges. However, a significant proportion of 29%, 54% and 15% of respondents clinically presented with depression, anxiety, and stress respectively. Furthermore, nearly 60% of respondents clinically presented with any form of the above mental disorders.

**Table 3:** Prevalence estimates of depression, anxiety, and stress among medical students during the COVID-19 pandemic, Tanzania, 2021 (n = 425). Significant values are in bold.

Rating	n (%)
Depression grade	
Normal	188 (44.24)
Mild	114 (26.82)
Moderate	99 (23.29)
Severe	15 (3.53)
Very severe	9 (2.12)
Clinically with depression	**123 (28.94)**
Anxiety grade	
Normal	151 (35.53)
Mild	44 (10.35)
Moderate	144 (33.38)
Severe	35 (8.24)
Very severe	51 (12.00)
Clinically with anxiety	**238 (54.12)**
Stress grade	
Normal	328 (77.18)
Mild	33 (7.76)
Moderate	38 (8.94)
Severe	16 (3.76)
Very severe	10 (2.35)
Clinically with stress	**64 (15.06)**
Clinically with any mental disorder	**249 (58.59)**

### Predictors of mental health disorders

Table 4 shows the results of a series of logistic regression models to examine the associations between selected independent variables and mental health disorders. For depression, model 1a and the corresponding model 1b that adjusted for all selected factors from model 1a, the odds of having depression were nearly six times higher for years four and five and two times higher for married/cohabiting respondents compared to their counterparts. For anxiety, model 2a and the corresponding model 2b that adjusted for all selected factors from model 2a, the odds of having anxiety were two times higher for married/cohabiting respondents and those from privately owned universities compared to their counterparts. For stress, model 3a and the corresponding model 3b that adjusted for all selected factors from model 3a, the odds of having stress were three times higher for respondents from privately owned universities compared to their counterparts. For having any mental disorders, model 4a and the corresponding model 4b that adjusted for all selected factors from model 4a, the odds for having any mental health disorders were almost three times higher for married/cohabiting respondents, two times higher for those from privately owned universities and those who are in clinical rotations compared to their counterparts.

**Table 4:** logistics regression models for factors associated with different types of mental health disorders among medical students during COVID-19 pandemic, Tanzania, 2021 (n = 425). Significant values are in bold.

Variable	Depression		Anxiety		Stress		Any MH	
	OR (95% CI)		OR (95% CI)		OR (95% CI)		OR (95% CI)	
	Model 1a	Model 1b	Model 2a	Model 2b	Model 3a	Model 3b	Model 4a	Model 4b
Age								
18–21	1.00	1.00	1.00	1.00	1.00	1.00	1.00	1.00
22–25	0.74 (0.33–1.67)	0.37 (0.12–1.08)	1.77 (0.81–3.90)	2.54 (0.71–9.13)	2.48 (0.57–10.81)	2.61 (0.54–12.51)	1.60 (0.74–3.45)	1.53 (0.44–5.35)
26–29	0.70 (0.29–1.70)	0.30 (0.09–1.01)	2.45 (1.05–5.74)	3.00 (0.72–12.43)	2.20 (0.47–10.29)	1.83 (0.33–10.16)	2.10 (0.91–4.84)	1.63 (0.40–6.60)
≥ 30	1.27 (0.42–3.83)	0.43 (0.10–1.77)	4.21 (1.33–13.30)	3.46 (0.59–20.18)	4.26 (0.78–23.44)	3.44 (0.50–23.65)	4.92 (1.45–16.73)	2.97 (0.46–19.27)
Year level								
First	1.00	1.00	1.00	1.00	1.00		1.00	1.00
Second	3.23 (0.83–12.58)	4.42 (0.96–20.34)	1.85 (0.67–5.13)	0.59 (0.12–2.82)	1.00 (0.27–3.75)		2.21 (0.80–6.15)	0.89 (0.19–4.16)
Third	3.33 (0.92–12.11)	3.96 (0.84–18.74)	2.44 (0.96–625)	0.82 (0.17–3.84)	1.54 (0.47–5.00)		3.27 (1.27–8.43)	1.52 (0.33–7.08)
Fourth	2.93 (0.82–10.43)	**5.99 (1.31–27.47)**	1.90 (0.77–4.69)	0.81 (0.16–3.94)	0.71 (0.22–2.33)		2.08 (0.84–5.16)	0.98 (0.20–4.73)
Fifth	2.33 (0.65–8.33)	**5.52 (1.18–25.81)**	1.63 (0.66–4.01)	0.34 (0.07–1.72)	0.51 (0.15–1.72)		2.07 (0.84–5.10)	0.58 (0.12–2.89)
Marital status								
Single	1.00	1.00	1.00	1.00	1.00	1.00	1.00	1.00
Married/cohabiting	1.78 (1.13–2.80)	**1.84 (1.08–3.15)**	2.23 (1.43–3.50)	**2.35 (1.72–2.76)**	1.49 (0.84–2.62)	1.10 (0.56–2.15)	2.57 (1.60–4.12)	**2.71 (1.88–3.33)**
University type								
Public	1.00	1.00	1.00	1.00	1.00	1.00	1.00	1.00
Private	1.71 (1.05–2.78)	1.46 (0.87–2.45)	2.99 (1.79–4.99)	**2.32 (1.20–4.50)**	2.81 (1.59–4.96)	**2.90 (1.60–5.27)**	1.94 (1.73–5.01)	**2.06 (1.04–4.09)**
Clinical rotation								
No	1.00		1.00	1.00	1.00	1.00	1.00	1.00
Yes	1.14 (0.72–0.80)		1.62 (1.07–2.46)	1.58 (0.68–3.71)	1.67 (0.89–3.14)	1.41 (0.70–2.86)	1.62 (1.07–2.46)	**1.70 (1.02–4.11)**
Vaccination status								
Unvaccinated	1.00		1.00	1.00	1.00		1.00	1.00
Vaccinated	0.99 (0.59–1.67)		1.47 (0.90–2.42)	1.35 (0.80–2.28)	1.32 (0.68–2.62)		1.42 (0.85–2.36)	1.26 (0.74–2.15)

## Discussion

In general, the academic life and job descriptions of medical students necessitate the utilization of patients who sometimes present with infectious conditions to comprehend diverse medical conditions and their appropriate management. This exacerbates their vulnerability to mental health disorders such as depression, anxiety, and stress. In addition, situations such as pandemic of highly infectious and fatal diseases such as COVID-19 may augment the detrimental effects experienced by medical students during their learning process^32,33^. Therefore, the current study aimed to assess the mental health status among medical students in Tanzania during the COVID-19 pandemic. The findings from the current study revealed high proportions (nearly 60%) of respondents had clinical symptoms related either to depression, anxiety, or stress. Furthermore, it revealed that being in the fourth/fifth year (clinical rotation), living with a partner, and studying in private universities were significant predictors of presenting symptoms related to mental health disorders.

The observed high proportion of symptoms related to mental health disorders among medical students in the current study is lower than that observed in other studies conducted in Egypt^32^ and Sudan^34^. The observed disparities in findings between current and previous studies might be due to differences in the type and number of tools used to assess symptoms of mental health disorders. The current study assessed depression, anxiety, and stress using single tool namely DASS-21, while the study in Egypt utilized two tools DASS-21 and the Impact of Event Stress Scale-Revised (IES-R). Hence, the aforementioned approach increased the sensitivity to identify individuals with symptoms related to mental health disorders. Furthermore, it is plausible that the disparity in findings between these studies may be attributable to the absence of lockdown as a measure to prevent the spread of COVID-19 infection in Tanzania, as compared to Egypt, Sudan, and other African nations. This assertion is substantiated by the findings of previous studies that have demonstrated that the implementation of a lockdown strategy to fight against COVID-19 infection has been associated with reported instances of mental health disorders such as depression, anxiety, and stress^35,36^. Furthermore, it is possible that the disparities may be attributable to variations in the socio-political context. The medical students in Sudan were subjected to social instability, economic suffering, and changes in the political environment. All of these situations, together with the COVID-19 pandemic, resulted in frequent and prolonged closures of public institutions including universities and colleges. The high proportion of symptoms related to mental health disorders among medical students in these studies calls on local authorities and international agencies to design appropriate immediate interventions for curbing this burden.

The current study shows that of the three assessed mental health disorders, the majority of medical students had symptoms related to anxiety (54%). This pattern is similar to that reported in previous studies conducted in Palestine, although it reported a high proportion of anxiety (89%)^37^ and Pakistani (88%)^38^. The patterns of anxiety being highly prevalent compared to depression and stress might be observed even in the general population. This has been demonstrated by previous systematic and meta-analyses, which observed similar patterns in the general population^39^. Furthermore, the current and previous studies utilized the psychometric scale DASS-21 to assess symptoms related to mental health disorders. As a results, they are more likely to capture a similar tendency irrespective of the settings or study populations. These findings suggest that anxiety is more prevalent than depression and stress not only among medical students or during COVID-19 but also among the general population and even before the pandemic.

The current study also assessed the factors associated with having symptoms of mental health disorders among medical students during the COVID-19 pandemic. The findings showed that being in clinical rotation during the pandemic was associated with having symptoms related to mental health disorders. In Tanzania, the majority of these students are in the fourth and fifth years of their studies, and as a result, they are six times more likely to experience depression compared to pre-clinical students (first to third year). Nonetheless, this observation contrasts with from the findings of previous study conducted among medical students in Peru, which revealed that pre-clinical students were more likely to experience symptoms related to mental health disorders^40^. This is supported by the findings from other study that suggested that mental health problems in medical students are more frequent during the early years of their schooling and decreases as their education progresses^41^. The observed differences may be due to differences in the medical school lifestyles between the study areas. In Tanzania, medical students are easily adapting the teaching styles during the pre-clinical that is not much different from high school education. However, previous studies have indicated that pre-clinical medical students may encounter certain challenges, possibly owing to relocation and a lack of medical school experience, which may have been negatively impacted by the COVID-19 pandemic, resulting in prominent symptoms related to mental health disorders even during the initial stage of their university schooling^40,42^.

Furthermore, the current study revealed that living with a partner (married/cohabiting) was significantly associated with having symptoms related to depression and anxiety. to the reason for this could be that being closer to someone such as a spouse increases the risk of COVID-19 transmission, as spouses spend more time together. This might have increased the fear of being contracted with this disease for medical students who were living with partners considering there was no lockdown in Tanzania during the pandemic. Therefore, were more likely to present with symptoms related to depression and anxiety during the COVID-19 pandemic compared to single individuals. This finding is supported by previous studies^43,44^ which suggested that living with close family members such as a spouse increases the risk of depression and anxiety among healthcare workers. However, these findings contradict those reported from other previous studies which suggested that single individuals were more likely to present with symptoms related to depression and anxiety^45,46^.

Last but not least, our study has highlighted that being medical students in private universities increased the likelihood of having symptoms related to anxiety and stress. This might be due to fear of freezing or lockdown which could have resulted in repayment of tuition fees which are nearly four times higher in private universities compared to public ones. Several previous studies have reported similar findings^47,48^.

To the best of our knowledge, this is the first study in Tanzania to assess mental health status among medical students who were required to continue learning even in a terrifying situation such as the COVID-19 pandemic. The inclusion of both private and public universities across all zones of Tanzania ensures that the collected data is representative. This implies a high degree of generalizability and accuracy in characterizing the mental health status among medical students during the COVID-19 pandemic in Tanzania.

Nonetheless, the cross-sectional nature of the study presents a limitation, as it failed to document whether the exposures occurred prior to the outcome, which could have affected the observed association. Furthermore, the utilization of the validated DASS-21 tool based on general populations of the United Kingdom (UK) possible might have introduced measurement variance owing to the absence of cross-cultural equivalence of the items. Therefore, it is important to interpret the present findings with caution. In addition, there might be possibility of misclassification bias due to the use of an arbitrary approach to dichotomize the outcome variables. This might have either under or overestimated the prevalence of outcome variables and their associations with independent variables.

## Conclusions

The findings from the current study indicate the need for regular checkups for medical students regarding their mental health status. Additionally, it recommends to the government and other stakeholders to establish mental health services within the universities to effectively mitigate the rising burden of mental health problems among universities in Tanzania and other countries with similar settings. Furthermore, medical students especially those in their clinical rotations (fourth and fifth year) should be given mental health education to expand their sense of self-esteem so that they can easily adapt in case they face difficult situations during their medical school programs such as the COVID-19 pandemic. This might be an effective way to manage symptoms related to mental health disorders among medical students in Tanzania.

### Supplementary Information

Supplementary Table 1.

## Data Availability

The dataset used for this study is restricted by the Ethical Research Committee of the University of Dodoma, as it contains sensitive patient information. However, it can be accessed upon reasonable request from the Directorate of Research Publication and Consultancy, University of Dodoma, P.O. Box 259, Dodoma, Tanzania (drpc@udom.ac.tz).

## Abbreviations

COVID-19: Coronavirus disease 2019
CUHAS: Catholic University of Health and Allied Sciences
DASS: Depression, anxiety, and stress scale
HKMU: Hubert Kairuki Memorial University
IQR: Interquartile range
MUHAS: Muhimbili University of Health and Allied Sciences
OR: Odds ratio
SFUCHAS: St. Francis University College of Health and Allied Sciences
UDOM: The University of Dodoma
UDSM: University of Dar es Salaam
WHO: World Health Organization
