# Supplementary material for: Mental health disorders among medical students during the COVID-19 pandemic in the area with no mandatory lockdown: a multicenter survey in Tanzania
Source: Sci Rep. 2024 Feb 11;14:3451. doi: 10.1038/s41598-024-53885-5 (PMC10859363; doi:10.1038/s41598-024-53885-5)
Supplement: Supplementary file 1 — Supplementary Table 1. [file 41598_2024_53885_MOESM1_ESM.docx]

**Supplementary Table 1 Testing for Reliability of DASS 21 items**

| **Variable** | **Cronbach alpha** | | | |
| --- | --- | --- | --- | --- |
|  | Depression  (7 items) | Anxiety  (7 items) | Stress  (7 items) | DASS 21  (21 items) |
| QD01 | 0.77 |  |  | 0.92 |
| QD02 | 0.74 |  |  | 0.92 |
| QD03 | 0.73 |  |  | 0.92 |
| QD04 | 0.72 |  |  | 0.92 |
| QD05 | 0.71 |  |  | 0.92 |
| QD06 | 0.72 |  |  | 0.92 |
| QD07 | 0.71 |  |  | 0.92 |
| **Cronbach alpha for depression 0.76** | |  |  |  |
| QA01 |  | 0.86 |  | 0.92 |
| QA02 |  | 0.84 |  | 0.92 |
| QA03 |  | 0.84 |  | 0.92 |
| QA04 |  | 0.83 |  | 0.91 |
| QA05 |  | 0.83 |  | 0.91 |
| QA06 |  | 0.84 |  | 0.92 |
| QA07 |  | 0.84 |  | 0.92 |
| **Cronbach alpha for depression 0.86** | | |  |  |
| QS01 |  |  | 0.85 | 0.92 |
| QS02 |  |  | 0.85 | 0.92 |
| QS03 |  |  | 0.85 | 0.91 |
| QS04 |  |  | 0.86 | 0.91 |
| QS05 |  |  | 0.84 | 0.92 |
| QS06 |  |  | 0.85 | 0.92 |
| QS07 |  |  | 0.85 | 0.92 |
| **Cronbach alpha for depression 0.87** | | | |  |
| **Overall Cronbach alpha for all DASS items 0.92** | | | | |
